# Supplementary material for: Vocalization during agonistic encounter in Mongolian gerbils: Impact of sexual experience
Source: PLoS One. 2022 Aug 2;17(8):e0272402. doi: 10.1371/journal.pone.0272402 (PMC9345364; doi:10.1371/journal.pone.0272402)
Supplement: Attachment — Submitted filename: Comments to the Author-PONE-D-21-38093.docx [file pone.0272402.s002.docx]

Comments to the Author

I have some major comments:

1) Why do intruders only use virgin gerbils ？
2) Whether or not there are difference between the structure of vocalizations before the attack and the after the attack? It might be possible that fighting has been expressed in voice before the beginning of physical conflict, which is a characteristic of vocalizations in the aggressive strategy.
3) It should be added to some explanations on the ecological function of the high- and low-frequency vocalizations related to non-agonistic and agonistic interactions in the discussion.

The minor comments:

1) Is it more appropriate to change the title to“ Vocalization during agonistic encounter in Mongolian gerbils：impact of sexual experience.”
2) Result Lines 3-7 should belong to the method behavior observation part，that is part of“same-sex resident-intruder test” , and it is recommended to adjust.
3) Page 23 line 13 “These results indicate that vocalizations….” should revised” These results indicated that vocalizations….”
