# Supplementary material for: Vocalization during agonistic encounter in Mongolian gerbils: Impact of sexual experience
Source: PLoS One. 2022 Aug 2;17(8):e0272402. doi: 10.1371/journal.pone.0272402 (PMC9345364; doi:10.1371/journal.pone.0272402)
Supplement: Attachment — Submitted filename: Comments to the Author-PONE-D-21-38093R1.docx [file pone.0272402.s004.docx]

Comments to the Author

This revised version has revised and supplemented the necessary information, and if the minor comments were revised, it could be considered to accept.

I think the descriptions of several behaviors that were scored should be more specific and detailed. We cannot define a target behavior with itself, for example, dig was defined by digging, and what is dig?.

| Jump | | Jumping vertically |
| --- | --- | --- |
| Move away | Moving away from the another | |
| Dig | Digging beddings on the floor | |
| Explore | Exploring the cage | |
| Stop | Stop moving | |
| Approach | Approaching within one body length of another | |

Maybe can refer to the reference: Hurtado-Parrado C, Gonzalez CH, Moreno LM, Gonzalez CA, Arias M et al., 2015. Catalogue of the behaviour of *Meriones unguiculatus* f. dom. (Mongolian gerbil) and wild conspecies, in captivity and under natural conditions, based on a systematic literature review. J Ethol 33:65–86
