# Supplementary material for: Vocalization during agonistic encounter in Mongolian gerbils: Impact of sexual experience
Source: PLoS One. 2022 Aug 2;17(8):e0272402. doi: 10.1371/journal.pone.0272402 (PMC9345364; doi:10.1371/journal.pone.0272402)
Supplement: Attachment — Submitted filename: Responses to reviewers.docx [file pone.0272402.s005.docx]

**Reviewer #1**

Thank you for your kind suggestions to improve this manuscript.

As detailed below, we have addressed all of the reviewer’s reservations.

The changes are highlighted in the marked text and “LXX” corresponds to lines in the marked text.

**Reviewer’s comment:**

L 30. We also confirmed two types of vocalizations during the encounters

Replace "two types" with "two groups of vocalizations" (as in L 176). Otherwise, types of vocalizations confuse with nine call types (see also L 256 and L 405).

**Author’s response:** We corrected as the reviewer suggested (L30).

**Reviewer’s comment:**

L 31. high-frequency (>24.6 kHz) and low-frequency (<24.6 kHz) with multiple harmonics

Delete "with multiple harmonics" after "low-frequency". The high-frequency vocalizations also have multiple harmonics. Create the spectrum up to 300 kHz, increase the intensity, and you will see them clearly.

**Author’s response:** We deleted "with multiple harmonics" (L31).

**Reviewer’s comment:**

L 106 Aggressive behaviors were initiated by residents in the vast majority (20/24) of tests

Why 24, not 26? You have in total 26 tests.

**Author’s response:** We corrected the number. The correct number is 20/26 (L106). Thank you for pointing this out.

**Reviewer’s comment:**

L 163. we identified nine calls

Replace "nine calls" with "nine call types"

**Author’s response:** We replaced it (L163).

**Reviewer’s comment:**

L 224 gerbil groups submitted to resident-intruder tests.

Replace "submitted to" with "during".

**Author’s response:** We replaced it (L224).

**Reviewer’s comment:**

L 254-256 the nine call types could be categorized into two groups. From the fitting functions of two normal distributions, the separation at 24.6 kHz was determined. Thus, for further analysis, we categorized the quantified nine calls of vocalizations into two types

Nine call types (L 254) cannot be categorized to the two call types. Replace with "we categorized the nine types of vocalizations into two groups"

**Author’s response:** We replaced it (L256).

**Reviewer’s comment:**

L 262-263 High-frequency syllables are represented by bluish symbols and low-frequency syllables

Replace "syllables" with "groups" (as earlier in L 176). Avoid synonyms.

**Author’s response:** We replaced it (L262-263).

**Reviewer’s comment:**

L 402-406 We found that gerbils emitted nine types of vocalizations during the resident-intruder test. The spectral shapes of these calls were basically consistent with those reported in previous studies [17, 18]. Alert calls described in the same study [17] were not observed in our resident-intruder tests. The nine calls are categorized into two types; high-frequency vocalization and low-frequency vocalization with harmonics.

Nine types of vocalizations (L 402) cannot be categorized to the two call types. Using consistently own terms throughout the text is important for understanding the content by the readers. Replace with "The nine call types are categorized into two groups"

**Author’s response:** We replaced it (L405-406).

**Reviewer’s comment:**

L 406 low-frequency vocalization with harmonics.

Delete "with harmonics". Presence of visible harmonics is not exclusively attributive to low-frequency vocalizations, but depends on the size of the used for analysis spectral window.

**Author’s response:** We deleted "with harmonics" (L406).

**Reviewer’s comment:**

L 444 resident intruder

Replace with resident-intruder

**Author’s response:** We replaced it (L444).

**Reviewer’s comment:**

L 443-456 This paragraph do not provides useful information and is very far from the results of this study. Delete it.

**Author’s response:** We think that these discussions are useful to consider the multiple aspects of behaviors observed during agonistic interactions. We also rewrote the paragraph to make this more related to the present study.

**Reviewer’s comment:**

L 464-467 However, given that low-frequency calls were recorded even during the period before the resident started attacking the intruder in MF-VF pairs (this was not observed in the other groups), it would be reasonable to estimate that the majority of low-frequency calls were emitted by MF residents.

This not an argument. Avoid speculations. In rodents, a lot of information comes from olfactory channel. It is easy to propose that VF-intruders placed on territory of MF-residents, perceive the smell of adult females and start calling of fear before the first aggressive interaction. A few more opposite arguments can be advanced.

**Author’s response:** We made the statement moderate. We think that estimations/assumptions belong to the discussion and there is no digital separation between estimations (or rational speculations) and argument.

**Reviewer’s comment:**

L 473-476 However, it remains unknown why MF gerbils (or MF-VF pair) emitted more low-frequency calls compared with those in the other three pairs. We hypothesize that vocalizations are influenced by hormonal changes induced by parenting experiences in female Mongolian gerbils.

In the preceding paragraph, you write that it is impossible to establish, who is calling the low-frequency calls, MF or VF. However, here you attribute the calls to one of females of the pair. Please re-write to make the content consistent with the text above.

**Author’s response:** We rewrote that (L474).

**Reviewer #2**

Thank you for your kind suggestion to improve this manuscript.

As detailed below, we have addressed the reviewer’s reservation.

**Reviewer’s comment:** I think the descriptions of several behaviors that were scored should be more specific and detailed. We cannot define a target behavior with itself, for example, dig was defined by digging, and what is dig?.

**Author’s response:** We updated the descriptions in the table.
